# Sleep Disturbance Trajectories in Critically Ill Children Post-ICU Discharge: A Longitudinal Observational Study

**DOI:** 10.3390/children13040568

**Published:** 2026-04-20

**Authors:** Wenchao Wang, Xiaorui Fan, Yuxia Yang, Jos M. Latour, Guoping Lu, Ying Gu

**Affiliations:** 1 Children’s Hospital of Fudan University, Shanghai 201102, China; wenchaowang_ww@fudan.edu.cn (W.W.);; 2 Shenzhen Children’s Hospital, Shenzhen 518026, China; 21211170015@m.fudan.edu.cn; 3 School of Nursing and Midwifery, Faculty of Health, University of Plymouth, Plymouth PL4 8AA, UK; jos.latour@plymouth.ac.uk

**Keywords:** children, parents, pediatric intensive care unit, trajectory, post-intensive care syndrome, sleep quality

## Abstract

**Highlights:**

**What are the main findings?**

• The peak of sleep disturbance of critically ill children occurred at 1-month after PICU discharge, and sleep duration and anxiety were recurring problems.
• Significant associations were found between sleep quality changes and the child characteristics, parental age, and clinical parameters; and the independent predictors for sleep quality were child age grade and surgical history.

**What are the implications of the main findings?**

• One month after PICU discharge may be a critical window to develop and implement targeted interventions to improve the sleep quality of PICU survivors.
• PICU nurses need to be aware of the importance of sleep for children in the PICU and collaborate with nurses from the pediatric wards to provide support to parents and children after hospital discharge.

**Abstract:**

**Background/Objectives**: Sleep disturbances have an impact on children’s physical and psychological development, yet little is known about the changes and factors influencing sleep after PICU discharge. To explore the trajectory of changes in sleep quality of critically ill children and to identify factors affecting sleep quality three months after Pediatric Intensive Care Unit (PICU) discharge. **Methods**: A longitudinal observation study was conducted between November 2022 and November 2023 at a tertiary children’s hospital. The Children’s Sleep Habits Questionnaire (CSHQ) was used at six time points: PICU-admission (T0), 1 week (T1), 2 weeks (T2), 1 month (T3), 2 months (T4), and 3 months (T5) after PICU discharge. The CSHQ is a 33-item parent-report outcome measure evaluating sleep problems. Total scores range between 33 and 99 points. A score of ≤41 indicates normal sleep; a score of >41 indicates sleep disturbance. Data were analyzed using the latent category growth model, univariate analysis, and multifactorial logistic regression. **Results**: Parents of 237 children completed all follow-up surveys. Prevalence of sleep disorders at T0-T5 of children with a score >41 were 46.5%, 83.5%, 69.7%, 54.3%, 50.2%, and 51.7%, respectively. General linear modeling revealed significant changes in CSHQ scores over time (F = 63.77, *p* < 0.05). The trajectories of identifying sleep changes were divided into three latent categories: High Sleep Disorder Group (n = 15, 6.33%), Moderate Sleep Disorder Group (n = 110, 45.2%), and No Sleep Disorder Group (n = 115, 48.52%). The trajectories were significantly different among preschool age, baseline sleep habit scores, surgery, and length-of-stay in pediatric wards (*p* < 0.05). The child’s age and surgical history were independent factors of sleep disturbance. **Conclusions**: The observed peak in sleep disturbances at 1-month post-PICU suggests that this period may be a critical window to develop and implement targeted interventions to improve sleep. The persistent sleep disorders highlight the need for long-term monitoring.

## 1. Introduction

Understanding long-term health outcomes of pediatric intensive care unit (PICU) patients is becoming increasingly important [1,2]. The concept of post-intensive care syndrome—pediatrics (PICS-p) has been expanded from the adult model to the pediatric population, taking into account the dynamic developmental state of children and their dependence on family and social structures [1]. The PICS-p encompasses physical, cognitive, emotional, and social symptoms that persist for weeks, months, or even years post-PICU discharge [3]. The survival rates of critically ill children have been improved due to the development of medical technology and the quality of care. However, many critically ill children may experience a range of problems across physical, psychological and social domains, such as anxiety, mental health and sleep disturbance [3,4].

Sleep disturbance is an increasingly recognized problem among children admitted to the PICU. Children in PICUs sleep 258 min less per night than those in general wards, and their nighttime awakenings are 2–6 times more frequent compared to children in general wards [5]. These sleep disturbances often persist long after hospital discharge. A long-term follow-up study of children with severe illnesses identified that sleep problems are significantly more prevalent in these children compared to their healthy counterparts, with 72% of patients at a higher risk of sleep disorders [6]. Additionally, several follow-up studies on children with traumatic brain injury (TBI) indicated that sleep disturbances in critically ill children often persist for years after discharge [7,8].

Children with poor sleep quality exhibited more emotional and behavioral problems compared to healthy children. Children are at a higher risk of developing sleep disorders during their stay in the PICU. This is often influenced by factors related to patients, environment, disease and ICU treatment. Studies have shown that sleep disturbance might weaken the child’s immune function, prolonging hospitalization, and increasing mortality [9,10]. However, sleep disturbance might also lead to decreased attention, memory loss, and emotional problems, which, in turn, affect patients’ future quality of life [11,12].

The post-intensive care syndrome—pediatric (PICS-p) dimensions of physical, cognitive, psychological and social health are intertwined and mutually influential, with each dimension potentially contributing to the occurrence of sleep disturbances after PICU discharge. The long-term impact of sleep disturbance on children’s health is becoming increasingly recognized as an area to increase the evidence to develop supportive interventions improving long-term health outcomes of PICU survivors. Therefore, the aim of our study was to explore sleep problems in the trajectory of changes in sleep quality of critically ill children within three months after PICU discharge and to identify factors affecting their sleep quality.

## 2. Materials and Methods

### 2.1. Design

This study was a longitudinal observational study using a validated questionnaire over six time points from PICU admission to three months after PICU discharge. The study is reported following ‘The Strengthening the Reporting of Observational Studies in Epidemiology (STROBE) Statement: guidelines for reporting observational studies’ [13]. The study protocol was approved by the hospital Institution Review Board (IRB number: (2022) 374). The study was prospectively registered on 17 November 2022 at ClinicalTrials.gov with the number NCT05863299. All parents provided written informed consent. The first part of the study, exploring sleep quality during PICU hospitalization, was published previously [14]. Here we report the second part of the study, the trajectory of sleep quality after PICU discharge.

### 2.2. Setting

The study was conducted at the PICU of a tertiary children’s hospital in China. The 55-bed PICU is a national referral center admitting critically ill children. The unit has six single-bedded rooms and multiple-bedded larger areas. Parents are allowed to visit their child during the day.

### 2.3. Participants

Children between 3 and 12 years of age and conscious at ICU admission, admitted to PICU for at least 48 h, were recruited between November 2022 and December 2023. Excluded were children with previously diagnosed psychiatric disorders such as cognitive dysfunction, brain injury, disorders of consciousness, epilepsy, obstructive sleep apnea–hypopnea syndrome, children with hearing impairments, and children with an ICU stay of >30 days. For children who experienced more than one PICU admission during the study period, only the first admission was considered. If a child was readmitted to the PICU after discharge, their follow-up data were censored at the time of readmission, and they were not included in later time point analyses.

The sample size was determined based on the Bayesian Information Criterion (BIC), which is frequently prioritized in model selection due to its higher sensitivity and accuracy in evaluating criteria [12]. To ensure the stability and reliability of parameter estimation, the BIC requires a sufficiently large sample size. Accordingly, a minimum sample size of n ≥ 200 was considered adequate. Accounting for an anticipated 15% attrition rate during follow-up, the final estimated sample size for this study was set at 240 participants. Ultimately, 237 cases were included in the final analysis.

### 2.4. Variables and Measurements

The child’s demographic data were collected, as well as the duration of nighttime noise (dB) and the duration of nighttime illumination (lux).

The Children’s Sleep Habits Questionnaire (CSHQ) [15] was used to measure quality of sleep. The CSHQ is a validated 35-item retrospective parent-report outcome instrument evaluating eight domains of sleep. The eight domains measure common sleep problems in children, including the following: D1: bedtime resistance (6 items; cutt-off > 10.84); D2: sleep anxiety (4 items; cut-off > 7.79); D3: sleep duration (3 items; cut-off > 5.27); D4: sleep disorder breathing (3 items; cut-off > 4.50); D5: parasomnias (7 items; cut-off > 10.61); D6: daytime sleepiness (8 items; cut-off > 15.24); D7: night wakings (3 itmes; cut-off > 5.29); and D8: sleep onset delay (1 item; cutt-off > 2.31). Scoring is based on 33 items because two items in two domains are identical. Scoring was based on a 3-point scale: Rarely (0–1 times/week), Sometimes (2–4 times/week), and Usually (5–7 times/week). The total score ranges from 33 to 99 points. Parents are asked to complete the CSHQ based on their child’s sleep behaviors during the past seven days. A total score of ≤41 indicates normal sleep, while a score of >41 indicates sleep disturbance. The reliability of CSHQ was α = 0.68 (children in community) and α = 0.78 (hospitalized children) [15]. The reliability of the translated Chinese CSHQ version was α = 0.73 [16].

Data collection included baseline and clinical characteristics such as demographics and treatment information. The baseline information, including T0 of the CSHQ, was collected with parents on admission to PICU, and clinical data were extracted from the electronic medical records. Parent provided sleep quality data over the first 3 months after PICU discharge through telephone follow-up by researchers. The data were collected at six time points: T0 (baseline, at PICU admission), 1 week (T1), 2 weeks (T2), 1 month (T3), 2 months (T4), and 3 months (T5) after PICU discharge.

### 2.5. Data Analysis

Data were analyzed using the statistical programs R (Version 4.3.2) and IBM-SPSS-20. Continuous variables were presented as mean and standard deviation (SD), while categorical variables were presented as frequencies and percentages. Comparisons of categorical data were conducted using the chi-square test. Incidence and time trend analysis of the CSHQ total scores and domains were calculated. Trajectory categories and characteristics were determined using a latent class growth model (LCGM). The baseline model began with a single category, incrementally increasing the number of categories in subsequent models. The optimal model was selected by comparing fit indices, balancing practical significance with statistical indicators, to determine the final number of trajectories and the characteristics of changes in children’s sleep patterns.

## 3. Results

**Figure 1:**
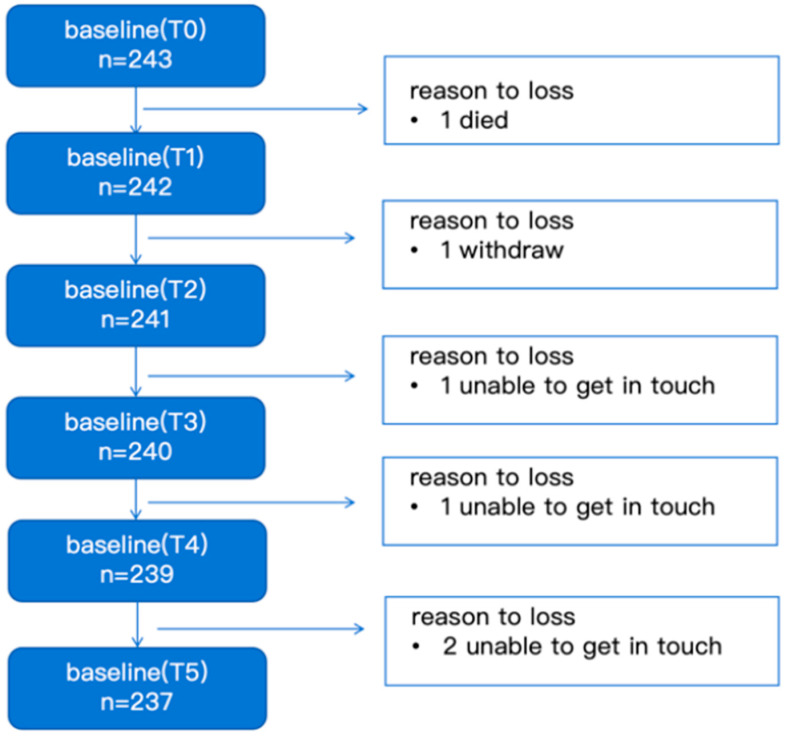
Flowchart of follow-up visit. The flowchart shows that 243 participants were enrolled at T0. Over the course of the study, participants were lost for various reasons: one participant died between T0 and T1, one withdrew between T1 and T2, one was unable to be contacted between T2 and T3, another was unable to be contacted between T3 and T4, and two were unable to be contacted between T4 and T5. The final number of participants retained at T5 was 237.

Parents of 243 children participated in the study. Of these, parents of 237 children completed all follow-up surveys, resulting in a lost-to-follow-up of 2.5%, including one death and five cases who withdrew from the study (Figure 1). The baseline characteristics of the participants are presented in Table 1. All children received invasive mechanical ventilation (i.e., endotracheal intubation or tracheostomy), and 21.8% of them received invasive respiratory therapy for more than 3 days. The mean duration of PICU stay was 7.13 days (SD = 6.98), and 113 (46.5%) children had a CHSQ score above 41. The most frequent surgical types were abdominal surgery, orthopedic surgery and neurosurgery. A complete breakdown of surgical categories is available in our previously published paper [14].

The prevalence of sleep disorders at T0~T5 were 46.50%, 83.50%, 69.70%, 54.30%, 50.20%, and 51.70%, respectively. The mean total scores of the CSHQ in T0~T5 were (40.17 ± 6.58), (47.61 ± 7.28), (44.26 ± 7.07), (41.53 ± 6.44), (40.72 ± 6.33), and (40.88 ± 6.35), respectively (Electronic Supplement Materials, Figure S1).

**Figure 2:**
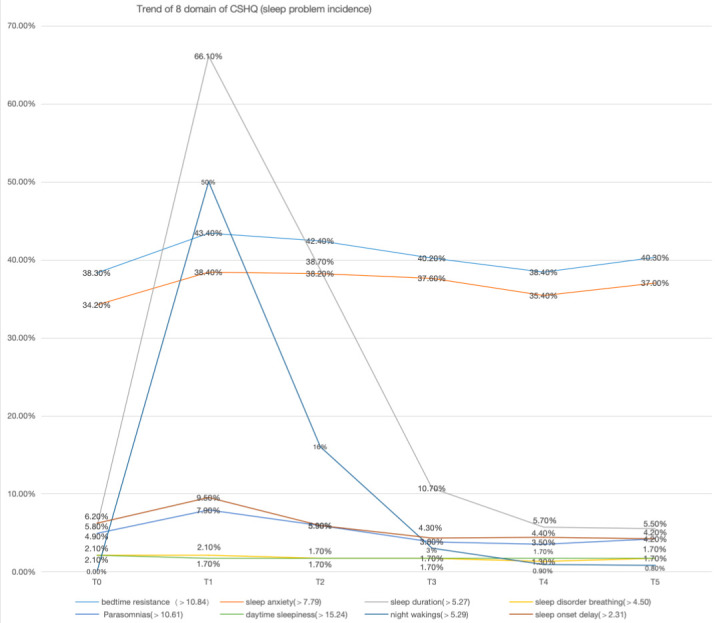
Trend of CSHQ 8 domain sleep problem in T0~T5. This figure presents the prevalence trends of eight sleep problem domains assessed via the Children’s Sleep Habits Questionnaire (CSHQ) across six time points (T0 to T5). Each line corresponds to a specific sleep domain, with the y-axis indicating the percentage of children experiencing clinically significant symptoms (exceeding domain-specific thresholds). Key observations include fluctuating prevalence rates across time points, with sleep duration (peaking at T2) and night waking (consistently high) showing notable trends. The data underscores heterogeneous sleep disturbances in this population, highlighting domains requiring targeted clinical attention.

The prevalence of the sleep duration domain reached 66.1% at time point T2, followed by night waking at 50% (Figure 2). Meanwhile, bedtime resistance and sleep anxiety showed average prevalence rates ranging from 34.2% to 43.4% across time points T0 to T5. Linear trends were tested through polynomial contrast analysis within repeated measures ANOVA, with Greenhouse–Geisser correction for sphericity violations. No significant time effects and linear trends were observed for D1 (F = 2.11; *p* = 0.08), D2 (F = 0.73; *p* = 0.57), D4 (F = 0.54; *p* = 0.62), and D6 (F = 1.21; *p* = 0.31), respectively; and significant temporal changes were detected in D3, D5, D7, and D8 (*p* < 0.05). Pairwise comparisons between time points showed no statistically significant differences (*p* < 0.05) between most time points for D5 and D6. (Electronic Supplement Materials—Figure S2).

The latent category growth model was used to analyze the model fit by extracting one to five categories sequentially, using the CSHQ score at six time points as the observables, set as the free estimation of the time parameter. The results of the study showed that as the number of categories increased, the Akaike Information Criteria (AIC), Bayesian Information Criteria (BIC) and Sample-Size-Adjusted Bayesian Information Criterion (SABIC) values decreased continuously, and the entropy value reached the maximum (Table 2).

**Figure 3:**
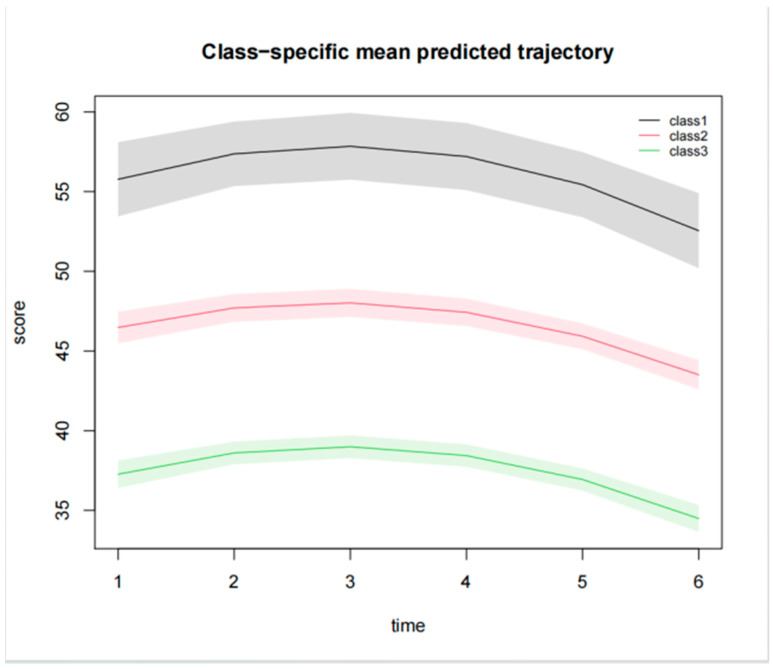
Trajectory of the latent class growth model for changes in sleep quality after PICU. The width of the shading represents the 95% confidence interval.

The latent trajectory analysis (Figure 3) revealed three distinct patterns of sleep quality evolution in pediatric critical care survivors. High Sleep Disorder Group (n = 15, 6.3%) was characterized by persistently elevated CSHQ score throughout observation and minimal improvement over three months follow-up; Moderate Sleep Disorder Group (n = 110, 45.2%) started at a level slightly higher than normal, increased rapidly over time, peaked at one month after the PICU (T3), and then slowly decreased and returned to normal levels; No Sleep Disorder Group (n = 115, 48.5%) with all the timeponits below clinical cut-off (CSHQ ≤ 41).

This figure displays the predicted trajectories of sleep quality scores over six time points (1 week, 2 weeks, 1 month, 2 months, 3 months, and 6 months post-discharge) for three latent classes identified via latent class growth analysis (LCGA). The three classes represent distinct sleep quality patterns: Class 1 (High Sleep Disorder Group, n = 15, 6.3%); Class 2 (Moderate Sleep Disorder Group, n = 110, 45.2%); Class 3 (No Sleep Disorder Group, n = 115, 48.5%).

Univariate analysis revealed significant associations with child characteristics, parental age and clinical parameters (Electronic Supplement Materials, Table S1). The multinomial logistic regression model identified independent predictors, including the child’s age, grade, and surgical history (Electronic Supplement Materials, Table S2).

## 4. Discussion

This study delineates the complex trajectory of sleep disturbances in pediatric survivors of critical illness, revealing three distinct sleep recovery patterns with significant clinical and research implications. The high retention rate (97.53%) across six follow-up points strengthens the validity of our findings. The observed sleep disturbance prevalence peaked at 83.5% one week post-ICU discharge (T1) and more than 50% of the child with the sleep disorder in three month (T4~T6), in accordance with the results of a systematic review, which showed the prevalence of sleep abnormalities in critically ill patients after hospital discharge was 50~66.7% (within 1 month), and 34–64.3% (>1–3 months) [18]. The mean total score of the CSHQ showed a distinct pattern of increasing and then decreasing over time. Regarding Figure 2, the finding that certain markers (e.g., night wakings) are particularly sensitive early after PICU discharge does not imply that they alone suffice for clinical follow-up. We advise against using only these sensitive indicators; rather, a multi-domain sleep assessment (including night wakings, sleep onset delay, daytime sleepiness, and night anxiety) should be applied, because the sensitivity of each marker changes with recovery time. These findings suggest that the evolution of sleep disturbances in pediatric critical care survivors is not merely monotonic but involves complex fluctuations. Future research should validate a core outcome set for post-PICU sleep disturbances.

Sleep duration and anxiety were the recurring problems in our study, especially in the first month after discharge from PICU. This aligns with a cohort study following up 151 children with brain injury after ICU discharge by using the CSHQ and showed that more than 50% of the children suffered from sleep–wake disturbances in the first months after discharge [19]. The high prevalence of sleep duration deficits (66.1% at T2) and night waking (50%) further corroborates the findings from Luther’s study [20], which highlighted sleep fragmentation as a common issue in critically ill children, especially after traumatic brain injury. Notably, the lack of significant time effects for domains like sleep anxiety contrasts with some studies, possibly due to differences in patient populations or measurement tools [18].

Using latent category growth modeling, we identified three distinct trajectories of sleep quality: High Sleep Disorder Group (6.33%), Moderate Sleep Disorder Group (45.2%), and No Sleep Disorder Group (48.5%). This categorization mirrors findings from Hassinge’s study, which reported heterogeneous sleep recovery patterns in pediatric ICU survivors, with a subgroup showing persistent disturbances [21]. The optimal three-category model aligns with methodological recommendations for latent growth models, which emphasize balancing model complexity and interpretability. The trajectory of the Moderate Sleep Disorder Group, peaking at one month post-ICU, supports the hypothesis that physiological and psychological stressors (e.g., prolonged mechanical ventilation and family separation) may intensify during early recovery.

Our multinomial logistic regression identified child age grade and surgical history as independent predictors of sleep disorder trajectories, consistent with Luther et al.’s study, which found that younger children and those undergoing surgery face higher risks of sleep disruption [19]. Nighttime anxiety and sleep disturbances were more common in younger children, particularly those aged 1–4 years. We speculate that separation trauma during the PICU stay may have played a role, as parents in our unit were allowed to visit only during the day. The absence of a parent at night could have heightened the child’s fear and insecurity, leading to prolonged nighttime anxiety after discharge. Future studies should examine the effect of liberalized parental presence on post-PICU sleep outcomes. The association between parental age and sleep quality highlights the role of caregiver stress in sleep outcomes, a theme supported by O’Meara et al., who emphasized parental coping strategies as critical mediators of child sleep health [22]. These findings underscore the need for family-centered interventions, such as sleep hygiene education and psychological support, to mitigate sleep disturbances.

Our study has some limitations. First, the reliance on parental-reported CSHQ scores may introduce recall bias, though this aligns with prior studies using similar tools. Future research could consider integrating objective activity measurement tools, such as actigraphy or wrist-worn accelerometers, to provide concurrent validation of sleep patterns and to capture circadian rhythm disruptions more precisely. Second, the follow-up study was conducted exclusively on patients in the PICU of a single tertiary children’s hospital in China, with a limited sample size that may limit the generalizability of the findings. Third, while the latent category model provided clinically meaningful groups, larger sample sizes are needed to validate these trajectories in diverse populations.

## 5. Conclusions

The observed peak in sleep disturbances at one month post-ICU suggests that the period may be a critical window for targeted interventions, such as behavioral sleep training or parental counseling. The identification of persistent sleep disorders highlights the need for long-term monitoring and referral to sleep specialists. And the association between surgical history and poor sleep outcomes supports the integration of perioperative sleep assessments into standard care protocols. Additionally, randomized controlled trials are needed to evaluate interventions tailored to specific trajectory groups (e.g., cognitive-behavioral therapy for the High Sleep Disorder Group). Finally, the inclusion of objective sleep measures (e.g., actigraphy) could strengthen the validity of self-reported data. Our findings underscore the importance of early, trajectory-tailored integrated sleep interventions, supported by long-term monitoring and objective sleep assessment, to mitigate post-PICU sleep disturbances and improve outcomes in critically ill children.

## Figures and Tables

**Table 1:** Baseline characteristics of the study participants (n = 243).

Baseline Characteristics	n (%)
Age grades	
Pre-school age (3–6 years old)	77 (31.7)
School age (7–12 years old)	166 (68.3)
Sex	
Male	142 (58.4)
Female	101 (41.6)
Type of residence	
Urban	147 (60.5)
Rural	96 (39.5)
Only child	
Yes	140 (57.6)
No	103 (42.4)
Primary caregiver	
Parents	177 (72.8)
grandparents	66 (27.2)
Previous PICU hospitalization experience	
Yes	105 (43.2)
No	138 (56.8)
Baseline-CSHQ scores	
<41	130 (53.5)
≥41	113 (46.5)
Primary diagnosis	
Neurological disease/tumor	80 (32.9)
Respiratory disease	59 (24.3)
Chemotherapy for malignant tumors	22 (9.1)
Accidental injury	23 (9.5)
Blood system disease	18 (7.4)
Digestive system disease	13(5.4)
Circulatory system disease	12(4.9)
Liver and kidney dysfunction diseases	8 (3.3)
Others *	8 (3.3)
Pediatric critical illness scores (PCIS) *	
>80	122 (50.2)
71~80	118 (48.6)
<70	3 (1.2)
Surgery history	
Yes	128 (52.7)
No	115 (47.3)
Days of continuous mechanical ventilation *	
<3 days	190 (78.2)
≥3 days	53 (21.8)
Days of continuous use of sedative-analgesic drugs	
<3 days	190 (78.2)
≥3 days	53 (21.8)
Constrain	
Yes	45 (18.5)
No	198 (81.5)
Cumulative invasive catheters (mean; SD)	3.71 ± 1.72
Number of fasting days (mean; SD)	1.18 ± 1.79
Night medical activities (mean; SD)	27.70 ± 7.51
Duration of nighttime noise exceeding 50 dB in hours (mean; SD)	4.32 ± 0.75
Duration of nighttime illumination exceeds 150 lux in hours (mean; SD)	2.50 ± 0.75
Length of ICU stay (mean; SD)	7.13 ± 6.98
Length of general ward stay (mean; SD)	5.16 ± 7.07

* PCIS-p = Pediatric critical illness scores [17]; * Mechanical ventilation refers to invasive positive pressure ventilation only. Children receiving only non-invasive ventilation (e.g., CPAP or BiPAP) are not included in this category.

**Table 2:** Results of model fitting for sleep habit scores in pediatric critically ill patients (n = 237).

Categories	Logic	AIC	BIC	SABIC	Entropy	Categorical Probability%				
1	−4085.05	8184.11	8208.38	8186.19	1.00	100.00				
2	−4077.48	8176.96	8215.10	8180.24	0.62	36.71	63.29			
3	−4063.98	8157.97	8209.99	8162.45	0.84	6.33	45.15	48.52		
4	−4059.89	8157.78	8223.67	8163.45	0.79	5.91	24.89	42.19	2.00	
5	−4052.37	8150.75	8230.51	8157.61	0.80	22.78	22.36	25.32	6.33	23.21

AIC = Akaike Information Criteria; BIC = Bayesian Information Criteria; and SABIC = Sample-Size-Adjusted Bayesian Information Criterion.

## Data Availability

The raw data supporting the conclusions of this article will be made available by the authors on request.

## Supplementary Materials

The following supporting information can be downloaded at: https://www.mdpi.com/article/10.3390/children13040568/s1. Figure S1: CSHQ total score means in T0~T5; Figure S2: Time trend analysis of eight domains of CSHQ (estimated marginal means); Table S1: Univariate analysis of trajectory categories of sleep quality changes in PICU (n = 237); Table S2. Multiple Logistic Regression Analysis of Trajectory Categories of Change in Sleep Quality After ICU.

## Abbreviations

The following abbreviations are used in this manuscript:

PICU	Pediatric Intensive Care Unit
CSHQ	Children’s Sleep Habits Questionnaire (CSHQ)
LCGM	Latent Class Growth Model
PICS-p	Post-Intensive Care Syndrome—Pediatric
PCIS	Pediatric Critical Illness Syndrome
