# Supplementary material for: Sleep Disturbance Trajectories in Critically Ill Children Post-ICU Discharge: A Longitudinal Observational Study
Source: Children (Basel). 2026 Apr 20;13(4):568. doi: 10.3390/children13040568 (PMC13114457; doi:10.3390/children13040568)
Supplement: Supplementary file 1 [file children-13-00568-s001.zip › children-4245312-supplementary.pdf]

Electronic Supplement Materials

Figure S1: CSHQ Total Score Means in T0~T5

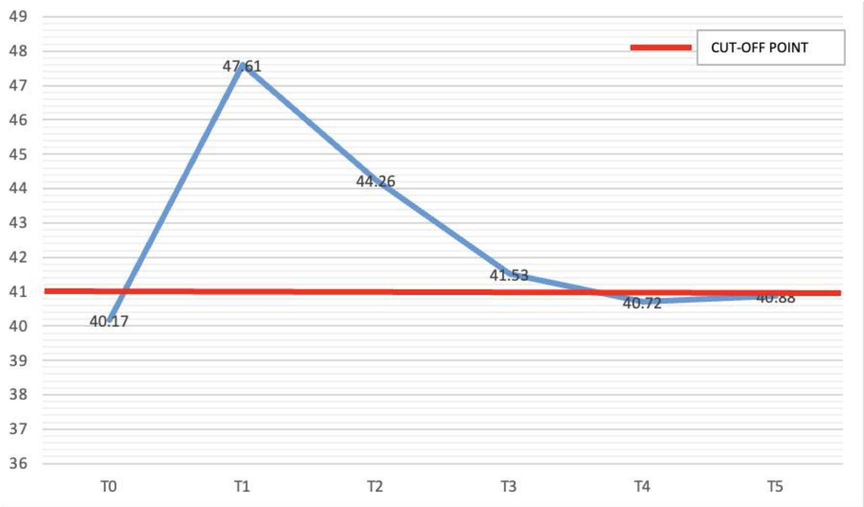

This line graph illustrates the mean total scores of the Children’s Sleep Habits Questionnaire (CSHQ) across six time points (T0 to T5) post-ICU discharge. The blue line represents the average CSHQ scores at each assessment, while the red horizontal line denotes the clinical cutoff point of 41, above which indicates potential sleep disturbances.

Figure S2: Time trend analysis of 8 domains of CSHQ (Estimated Marginal Means)

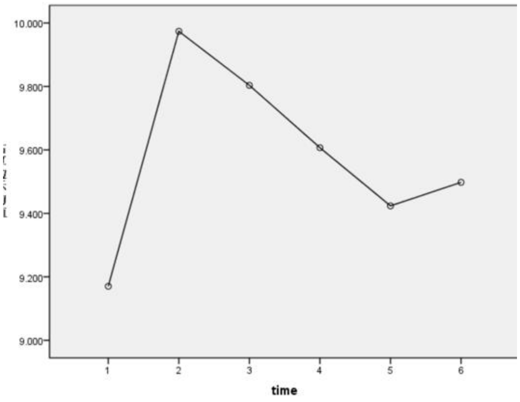

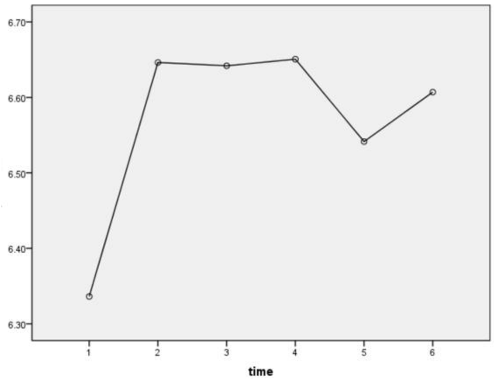

D1: Sleep resistance

D2: Sleep anxiety

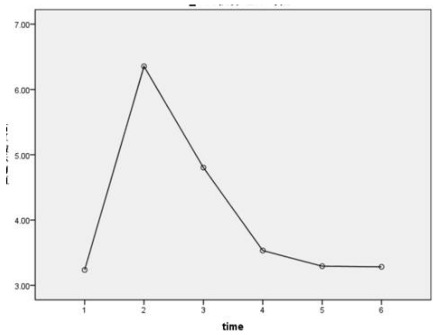

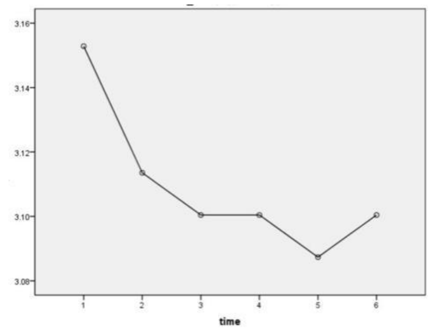

D3: Sleep duration

D4: sleep disorder breathing

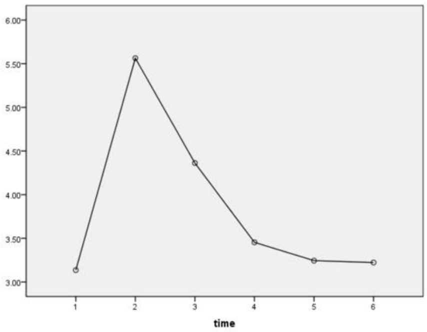

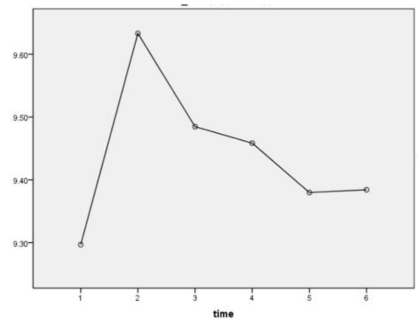

D5: Parasomnias

D6: daytime sleepiness

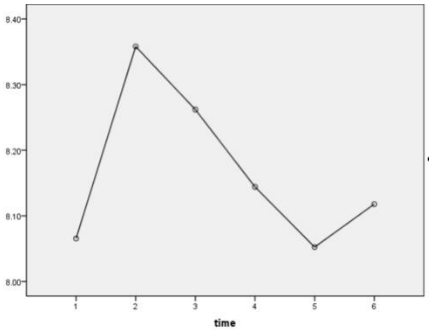

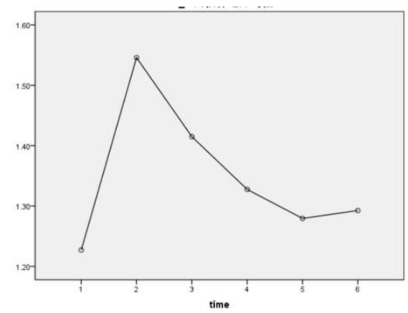

D7: night wakings

D8: sleep onset delay

D1-D8 are the right domain of CSHQ; Time 1-6=To-T5

Table S1: Univariate analysis of trajectory categories of sleep quality changes in PICU(n=237)

Categories		Class 1 (n=15)	Class2(n=107)	Class 3(n=115)	P-Value
Age grades (%)	Preschool-age	6 (40.0)	58 (54.2)	11 (9.6)	<0.001
	School-age	9 (60.0)	49 (45.8)	104 (90.4)	
Gender (%)	Female	4 (26.7)	46 (43.0)	49 (42.6)	0.471
	Male	11 (73.3)	61 (57.0)	66 (57.4)	
Only child (%)	Yes	5 (33.3)	65 (60.7)	68 (59.1)	0.126
	No	10 (66.7)	42 (39.3)	47 (40.9)	
Inhabitation (%)	Rural	6 (40.0)	37 (34.6)	49 (42.6)	0.469
	Urban	9 (60.0)	70 (65.4)	66 (57.4)	
Mother's age * (years)		34.33 (5.5)	33.06 (4.5)	37.27 (4.7)	<0.001
Father's age* (years)		37.47 (8.6)	34.71 (4.8)	38.71 (5.2)	<0.001
Primary caregiver (%)	Parents	12 (80.0)	80 (74.8)	80 (69.6)	0.550
	Grandparents	3 (20.0)	27 (25.2)	35 (30.4)	
Previous ICU hospitalization experience (%)	Yes	10 (66.7)	48 (44.9)	44 (38.3)	0.099
	No	5 (33.3)	59 (55.1)	71 (61.7)	
Baseline CSHQ score*		53.00 [47.0, 56.5]	42.00[35.5,45.0]	36.00[33.0,42.0]	<0.001
ICU days of hospitalization*		2.00 [1.0, 7.5]	5.00 [2.00, 9.50]	6.00 [3.00, 10.00]	0.018
General ward hospitalization days*		8.00 [6.0, 13.50]	4.00 [0.00, 8.50]	2.00 [0.00, 7.00]	0.006
Single Ward (%)	Yes	3 (20.0)	16 (15.0)	13 (11.3)	0.546
	No	12 (80.0)	91 (85.0)	102 (88.7)	
Constrain (%)	Yes	4 (26.7)	23 (21.5)	18 (15.7)	0.398

	No	11 (73.3)	84 (78.5)	97 (84.3)	
Bed posture (%)	Active	13 (86.7)	89 (83.2)	97 (84.3)	0.931
	Reactive	2 (13.3)	18 (16.8)	18 (15.7)	
PCIS (%)	71~80	10 (66.7)	53 (49.5)	56 (48.7)	0.417
	< 70	5 (33.3)	54 (50.5)	59 (51.3)	
Cumulative number of tubes*		4.00 [3.0, 5.0]	4.00 [2.0, 5.0]	3.00 [2.0, 5.0]	0.722
Number of days of fasting *		0.00 [0.0, 0.5]	1.00 [0.0, 2.0]	1.00 [0.0, 2.0]	0.065
Days of continuous mechanical ventilation (%)	< 3	12 (80.0)	81 (75.7)	93 (80.9)	0.638
	≥3	3 (20.0)	26 (24.3)	22 (19.1)	
Days of continuous diuretic use (%)	< 3	12 (80.0)	96 (89.7)	110 (95.7)	0.056
	≥3	3 (20.0)	11 (10.3)	5 (4.3)	
Days of continuous use of hormonal drugs (%)	< 3	14 (93.3)	90 (84.1)	92 (80.0)	0.383
	≥3	1 (6.7)	17 (15.9)	23 (20.0)	
Days of continuous use of sedative-analgesic drugs (%)	< 3	14 (93.3)	84 (78.5)	90 (78.3)	0.383
	≥3	1 (6.7)	23 (21.5)	25 (21.7)	
Surgical history (%)	Yes	14 (93.3)	58 (54.2)	53 (46.1)	0.002
	No	1 (6.7)	49 (45.8)	62 (53.9)	

CLASS1= High Sleep Disorder Group; CLASS2= Moderate Sleep Disorder Group;CLASS3= No Sleep Disorder Group

Table S2. Multiple Logistic Regression Analysis of Trajectory Categories of Change in Sleep Quality After ICU

Categories	Class1 vs Class3					Class2 vs Class3				
	B	SE	Wald	P	OR (95% CI)	B	SE	Wald	P	OR (95% CI)
Baseline CSHQ score*	0.287	0.06	22.923	<0.001	1.333(1.185-1.499)	0.094	0.028	10.893	0.001	1.099(1.039-1.162)
General ward hospitalization days	0.124	0.045	7.788	0.005	1.133(1.038-1.236)	0.068	0.026	6.561	0.01	1.07(1.016-1.127)
Preschool-age	2.736	0.856	10.226	0.001	15.426(2.884-82.517)	2.237	0.442	25.596	<0.001	9.368(3.937-22.287)
School-age					Ref					Ref
Surgical history	-2.304	1.137	4.11	0.043	0.1(0.011-0.926)	-0.468	0.362	1.673	0.196	0.626(0.308-1.272)

CLASS1= High Sleep Disorder Group; CLASS2= Moderate Sleep Disorder Group;CLASS3= No Sleep Disorder Group
